# Draft Genome Sequence of a Harveyi Clade Bacterium Isolated from Lolliguncula brevis Squid

**DOI:** 10.1128/MRA.00078-20

**Published:** 2020-02-20

**Authors:** Alecia N. Septer, Lauren Speare, Collin K. Coleman, Stephanie Smith, Coby Dorsey, Travis Wilson, Scott M. Gifford

**Affiliations:** a Department of Marine Sciences, University of North Carolina, Chapel Hill, North Carolina, USA; b Department of Environmental Sciences and Engineering, University of North Carolina, Chapel Hill, North Carolina, USA; Georgia Institute of Technology

## Abstract

*Vibrio* species of the Harveyi clade are commonly found in free-living and host-associated marine habitats. Here, we report the draft genome sequence for a Harveyi clade bacterium, *Vibrio* sp. strain LB10LO1, which was isolated from the Atlantic brief squid Lolliguncula brevis.

## ANNOUNCEMENT

Bacteria within the genus *Vibrio* are widely distributed throughout marine systems where they inhabit both nutrient-rich and oligotrophic environments (1). Within the *Vibrio* genus, members of the Harveyi clade include 11 closely related species that are commonly found in surface waters, in marine sediments, and as pathogens and commensals of both vertebrates and invertebrates (2–13). Moreover, these species serve as important model organisms for studying biofilm formation, bioluminescence, and quorum sensing (14).

Here, we introduce the genome sequence of a Harveyi clade bacterium, *Vibrio* sp. strain LB10LO1, which was isolated from a wild-caught Atlantic brief squid, Lolliguncula brevis. In the summer of 2017, L. brevis squid were collected from the bycatch of a trawl off the coast of Morehead City, North Carolina. A deceased animal was immediately washed with filter-sterilized instant ocean. Tissue within the mantle cavity was removed, homogenized, and plated directly onto Luria-Bertani with added salt (LBS) agar (15). Cultivation plates were incubated at 24°C overnight, and a brightly luminescent colony was picked and restreaked for purification, resulting in strain LB10LO1. The initial phylogeny of this isolate was determined using analysis of the *hsp60* and *toxR* sequences (16, 17), which suggested that LB10LO1 is a Vibrio campbellii species within the Harveyi clade. Because recent studies have shown that whole-genome comparisons are the best way to confirm phylogeny among Harveyi clade members (2), we sought to sequence the genome of LB10LO1 to determine how this isolate relates to other species within the Harveyi clade.

A single LB10LO1 colony was picked and streaked onto LBS agar plates and incubated overnight at 24°C. Genomic DNA was extracted from this clonal bacterial growth with a Zymo DNA miniprep kit, and the quantity and quality were determined using an Eppendorf BioSpectrometer. Library preparation was performed using a TruSeq DNA kit (Illumina, San Diego, CA, USA), following the manufacturer’s protocol. The library was sequenced using the MiSeq Illumina platform and 300-bp paired-end reads at the University of North Carolina (UNC) High-Throughput Genomic Sequencing Facility, resulting in a total of 1,798,287 paired reads. Raw reads were trimmed using Trimmomatic (18) using a 10-bp sliding window average, Phred score threshold of 20, and minimum read length of 50 nucleotides (nt). Paired reads were assembled using PEAR (default settings) (19). The remaining sequences were assembled using SPAdes (default settings) (20); sequences were annotated via the Prokka Prokaryotic Genome Annotation Pipeline, BlastKoala within the KEGG platform, and BioCyc (21).

The final draft genome of LB10LO1 is 5,515,790 bp long in 90 contigs (>1,000 bp), with a G+C content of 45.45%, 97-fold genome coverage, and an *N*_50_ score of 165,319 bp. A total of 4,995 DNA coding regions were identified, including 4,891 encoding proteins and 104 encoding RNAs. Prokka produced annotated functions for 3,127 of the proteins, while the other 1,764 proteins were assigned as hypothetical. Finally, a MiGA (22) and Genome Taxonomy Database (GTDB) (23) analysis of the LB10LO1 genome determined that the most closely related genomes in the database were Vibrio campbellii isolates, with an average nucleotide identity (ANI) of >96%.

### Data availability.

This genome sequence is available in GenBank under the BioProject number PRJNA602499; the Illumina reads are available in the SRA under accession number SRX7614634. Cultures of LB10LO1 are available upon request.
